# Brain metastases: It takes two factors for a primary cancer to metastasize to brain

**DOI:** 10.3389/fonc.2022.1003715

**Published:** 2022-09-28

**Authors:** Dingyun Liu, Jun Bai, Qian Chen, Renbo Tan, Zheng An, Jun Xiao, Yingwei Qu, Ying Xu

**Affiliations:** ^1^ Center for Cancer Systems Biology, China-Japan Union Hospital of Jilin University, Changchun, China; ^2^ College of Computer Science and Technology, Jilin University, Changchun, China; ^3^ School of Artificial Intelligence, Jilin University, Changchun, China; ^4^ Computational Systems Biology Lab, Department of Biochemistry and Molecular Biology and Institute of Bioinformatics, The University of Georgia, Athens, GA, United States

**Keywords:** brain metastasis, metabolic reprogramming, brain microenvironment, seed-soil hypothesis, blood-brain barrier

## Abstract

Brain metastasis of a cancer is a malignant disease with high mortality, but the cause and the molecular mechanism remain largely unknown. Using the samples of primary tumors of 22 cancer types in the TCGA database, we have performed a computational study of their transcriptomic data to investigate the drivers of brain metastases at the basic physics and chemistry level. Our main discoveries are: (i) the physical characteristics, namely electric charge, molecular weight, and the hydrophobicity of the extracellular structures of the expressed transmembrane proteins largely affect a primary cancer cell’s ability to cross the blood-brain barrier; and (ii) brain metastasis may require specific functions provided by the activated enzymes in the metastasizing primary cancer cells for survival in the brain micro-environment. Both predictions are supported by published experimental studies. Based on these findings, we have built a classifier to predict if a given primary cancer may have brain metastasis, achieving the accuracy level at *AUC* = 0.92 on large test sets.

## 1 Introduction

The general understanding about cancer is that given time, a vast majority of cancer biomasses will migrate and then metastasize to distal locations. Among the most observed metastasized organs are bone (accounting for 20% of all the distally metastasized cancers), brain (10% of the metastases), liver (20% of the metastases), and lung (15% of the metastases) (1). While multiple proposals have been made regarding the main factors that dictate where a primary cancer may metastasize to in the past 100+ years (2), it remains elusive about what molecular factors may determine the final destinations of a metastasizing cancer. Among the proposals, the *seed-soil hypothesis* made by Stephan Paget over 130 years ago remains a popular one, which essentially states: a primary cancer may metastasize to any organ *via* the circulatory systems, and it is the level of match between the functional capabilities (the seed) and the characteristics of the destination site (the soil) that dictate whether the newly arrived cells can survive the new environment (3). Another main proposal different from the seed-soil hypothesis is the anatomical-mechanical-based theory of metastasis, which lays the emphasis on the relevance of the vascular connections between the primary tumor and the target organ of metastasis (4).

The main question we address here is: what molecular factors may dictate if a primary cancer can metastasize to brain and thrive as a secondary tumor? The state of the art of understanding about brain metastases is that it requires at least two conditions, namely (a) the tumor cells should be able to cross the blood-brain barrier (BBB), possibly the most challenging step for tumor cells metastasizing into brain (5, 6), and (b) the brain-metastasized tumor cells must be able to adapt to the brain microenvironment to survive and thrive (7, 8). Following this guiding information, a number of studies have been published regarding the genomic and transcriptomic characteristics tumors relevant to (a) and (b). For example, (i) genetic mutations in *COX2*, *EGFR*, *HBEG*, *ST6GALNAC5* (9), and *MMP2*, *3*, *9* are found to be associated with the increased ability to penetrate the BBB (6, 10), particularly for breast cancer, the most studied primary cancer type in relevance to brain metastasis; and (ii) enhanced expressions of *IL1b*, *HES5* (11), *PCDH7* (12), γ-aminobutyric acid-related genes (13), and the activated Notch signaling pathway (7) can increase the ability of the arriving cancer cells to survive in the brain microenvironment.

We have conducted a computational study here aiming to elucidate the common and distinguishing features of primary cancer cells of different types having brain metastases. To do this, we have retrieved the transcriptomic data of all tissue samples of 22 cancer types from the TCGA database, 44 primary cancer samples known to have metastasized to brain, and 529 primary cancer samples known to have metastasized to other organs. We have then carried out a classification analysis focused on the physical features of the transmembrane proteins that are differentially expressed in primary cancers metastasized to brain *vs.* those to other organs. Then a second classification analysis is conducted for the same problem but utilizing expressed intracellular enzymes. Together the two classification analyses provide novel insights about the molecular factors that will enable metastasizing cancer cells to cross the BBB and that help the metastasizing cells to survive and thrive in the brain microenvironment.

Our predictions are largely supported by published studies. The insights gained here have considerably extended the state-of-the-art understanding about brain metastasis. In addition, these insights generalize the seed-soil hypothesis by Stephan Paget as the study shows that it requires more than just the level of match between the seed and the soil as the ability to get to the destination is an independent and a key factor. The tools developed here could be used to predict the potential for brain metastasis for given transcriptomic data of primary cancers.

## 2 Materials and methods

### 2.1 Data

#### 2.1.1 Gene expression data

RNA-seq data of 573 primary tumor samples of 22 cancer types, known to have metastasized, are retrieved from TCGA (Read-count and TPM values) (14). Of these, 44 have brain metastasis, denoted as BMPs (brain-metastasized primary tumors) and 529 have metastasized to bone, liver, and lung but not brain, denoted as NBMPs (non-brain-metastasized primary tumors). The NBMP samples are further divided into three subgroups according to the destination organ of the metastasis, denoted by NBMP-bone (113 samples), NBMP-liver (215 samples), and NBMP-lung (261 samples), respectively. In addition, 146 samples of primary brain cancer: glioblastoma multiforme (TCGA-GBM) are also downloaded from TCGA and used for comparison purposes. The detailed information about these samples is given in **Supplementary Table S1**.

#### 2.1.2 Transmembrane proteins and enzymes

The amino acid sequences in the extracellular regions of each plasma transmembrane protein are collected from Uniprot (15). The 2,779 human enzyme genes are collected from BioCyc (16). The detailed reaction information catalyzed by each enzyme is retrieved from Uniprot, whose functional information is collected from Human Protein Atlas (17) and GeneCards (18).

### 2.2 Methods

#### 2.2.1 Gene-expression analysis tools

Normalization of the RNA-seq data from different sources is done using Recount3 (19). The R function “DESeq” in package “DESeq2” is used for calculation of the differentially expressed genes (DEGs) between two group of samples. For read-count data, genes with |log2 *FC*| ≥ 1 having *P*-values< 0.05 are considered as DEGs, with *FC* being for fold change.

Function “corr.test” in the R package “psych” is used to calculate the Pearson correlation coefficient (*PCC*) between the expression profiles of two genes. Expression pairs with *PCC* > 0.6 having *P*-value< 0.0001 are deemed as being statistically co-expressed. The R function “GSVA” in package “GSVA” (20) is used to estimate the expression level of a gene set. This function is utilized to estimate the level of Fenton reaction using a set of 64 genes obtained from (21) (see Section 3.2)

#### 2.2.2 Pathway enrichment

Pathway enrichment is conducted over a given set of genes against three databases: KEGG (22), REACTOME (23), and GO Biological Process (24). Enriched pathways with *P*-value< 0.0001 are utilized in our analyses.

#### 2.2.3 Binary classification

Function “SVC” in Python is used to build a support vector machine (SVM)-based classifier to classify between the BMP and NBMP samples in terms of the provided data, including gene expressions. A Gaussian kernel is applied, with the parameter “class_weight” set to “balanced”. Before the classification analysis, function “RFE”, for executing the support vector machine-recursive feature elimination (SVM-RFE) algorithm, is used to rank the contribution by each enzyme gene to the BMP/NBMP classification result and to select the most contributing factors for the final classification. A five-fold cross validation is utilized to validate the classification model. Receiver operating characteristic (ROC) curves and the area under the curve (*AUC*) are used to evaluate the classification performance.

## 3 Results

Two key characteristics, namely specific physical features of the extracellular components of the expressed transmembrane proteins and the activity levels of selected intracellular enzymes, are found to be able to well distinguish between cancers that can metastasize to brain and those that cannot. Details follow.

### 3.1 BMPs share common and distinguishing characteristics in transmembrane proteins

Our hypothesis is that cancer cells that metastasize to brain must be able to go through the BBB. Published studies have shown that it is relatively easy for lipophilic molecules with low molecular weights and overall positive charges to cross the BBB (25–27). The reason for such results is that all cells’ plasma membrane, including the BBB endothelial cells, is lipophilic and negatively charged; and the tight junctions connecting such cells generally permit only small molecules (25–27). However, little is known about what characteristics a cell must have for it to go through the BBB. Our goal here is to identify features that can distinguish primary cancer cells that metastasize to brain vs. those that cannot, focusing on cell-surface proteins.

We have examined all the differentially expressed genes between BMP and NBMP samples (**Table S2A**), totaling 100 transmembrane proteins with 97 being downregulated and three upregulated in BMP vs. NBMP, as shown in **Table S2B**. We have then calculated the following features of the extracellular components of each such protein: the number of charges, the total molecular weight, and the hydropathy index, as defined in (1) - (5):


(1) $$EP(i)=N_{R}(i)+N_{H}(i)+N_{K}(i)$$

(2) $$EN(i)=N_{D}(i)+N_{E}(i)$$

(3) EC(i)=EP(i) - EN(i)

(4) EW(i)=W·N(i)

(5) EH(i)=H·N(i)

where *EP*(*i*) denotes the number of positive charges in the extracellular component of the *i* ^th^ transmembrane protein (NOTE: subsequences with< 10 amino acids are omitted since their locations are too close to the cell membrane to be useful for the classification, which is determined empirically), and *N_R_* (*i*), *N_H_* (*i*) and *N_K_* (*i*) represent the numbers of the amino acids arginine, histidine, and lysine in the extracellular parts of the protein, respectively, covering all amino acids each carrying a positive charge (**Table S2C**); similarly, *EN*(*i*) denotes the number of negative charges in the extracellular space of the *i* ^th^ transmembrane protein; and *N_D_* (*i*) and *N_E_* (*i*) are for the numbers of aspartate and glutamate, respectively, each carrying a negative charge; *EW*(*i*) denotes the total molecular weight of the extracellular components in the *i* ^th^ transmembrane protein, with vector ***W*** representing the molecular weight for each of the 20 amino acids (**Table S2C**), vector ***N*** (*i*) being the number of each of the 20 amino acids in the extracellular parts of the *i* ^th^ protein, and “·” for the inner product; and similarly, *EH*(*i*) represents the total extracellular hydropathy index of the *i* ^th^ transmembrane protein, with vector ***H*** being the hydropathy index for each of the 20 amino acids (**Table S2C**). The calculation results for the two classes of samples are shown in **Table S2B** and **Table 1**.

**Table 1:** Statistics of the extracellular physical characteristics of differentially expressed transmembrane proteins between BMPs and NBMPs.

Transmembrane protein	The number of proteins with *EC* > 0	The number of proteins with *EC*< 0	The number of proteins with *EH* > 0	The number of proteins with *EH*< 0	Median of *EW* (Da)
**Upregulated** **(BMP vs. NBMP)**	1	1	1	2	1.1282e^04^
**Downregulated** **(BMP vs. NBMP)**	34	51	11	85	2.7368e^04^

We note from **Table 1** that more than 50% (51/97) of the downregulated transmembrane proteins in BMP have negative total charges in their extracellular region, while 35% (34/97) and 12% (12/97) of them have positive and neutral total charges, respectively. In addition, more than 85% (85/97) of the downregulated proteins have negative *EH* values. Furthermore, the median *EW* value of the downregulated proteins is more than 20,000 Da, which is more than twice of that of the upregulated transmembrane proteins. These results indicate that BMP cells tend to have reduced or even inhibited cell-surface molecules with negative charges, of hydrophilic, and higher molecular weights in their expressed transmembrane proteins.

It is noteworthy that only three of the 100 differentially expressed transmembrane proteins are upregulated in BMP vs. NBMP: *DLL3*, *OPRD1*, and *PLP1*, where both *DLL3* and *OPRD1* play inhibitory roles in neurogenesis and neural activities, and *PLP1* is a proteolipid protein that can covalently link with lipids (17).

Overall, the above data suggest that BMPs tend to reduce the physical characteristics that prevent cells from going through the BBB (25–27). Our pathway enrichment analyses over the 97 downregulated transmembrane proteins reveal that the most suppressed functions are transporters and channels (60/116 pathways), followed by cell-cell adhesion and communications (19/116 pathways), and complex neural functions such as synapse activities and muscle contraction (15/116 pathways), accounting for over 81% of all the repressed pathways (**Table S2D**). As a summary, (i) cancers tend to suppress cell polarity genes, which include the transportation system and cell-cell adhesion (28) and; (ii) the synapse activity and muscle contraction (28, 29), hence consistent with the above feature-based analyses as well as the published studies (25–27).

Based on the analysis results, we have calculated the following four values as the main features for the BMP vs. NBMP classification:


(6) $$S_{\text{EP}}=\sum_{i=1}^{100}\left(P_{i}\times EP(i)\right)$$

(7) $$S_{\text{EN}}=\sum_{i=1}^{100}\left(P_{i}\times EN(i)\right)$$

(8) $$S_{\text{EW}}=\sum_{i=1}^{100}\left(P_{i}\times EW(i)\right)$$

(9) $$S_{\text{EH}}=\sum_{i=1}^{100}\left(P_{i}\times EH(i)\right)$$

where *S_EP_* and *S_EN_* denote the total extracellularly positive and negative charges carried by the 100 differentially expressed transmembrane proteins, respectively, with *P_i_* being the gene expression of the *i* ^th^ transmembrane protein; and *S*

*_EW_* and *S_EH_* are for the total molecular weight and hydropathy index values of the 100 proteins, respectively. The medians of *S_EP_*, *S_EN_*, *S_EW_*, and *S_EH_* for BMP, NBMP, NBMP-bone, NBMP-liver, and NBMP-lung samples are given in **Table S2E**, showing that the BMP samples have the lowest median value for all of the four features, among the five groups, and hence indicating that each of the selected physical characteristics is highly discerning in separating BMPs from each of the three NBMP groups. In addition, the median *S_EP_* is higher than that of *S_EN_* for BMP samples, indicating the 100 transmembrane proteins have a positive total charge on the extracellular components of BMP cells, which is different from that of each NBMP subgroup. The median of the four features on GBM samples show patterns similar to those of NBMPs rather than BMPs. This is presumably because the primary brain cancer cells grow directly in brain central nervous system (CNS) and do not need to cross the BBB.

**Figure 1:**
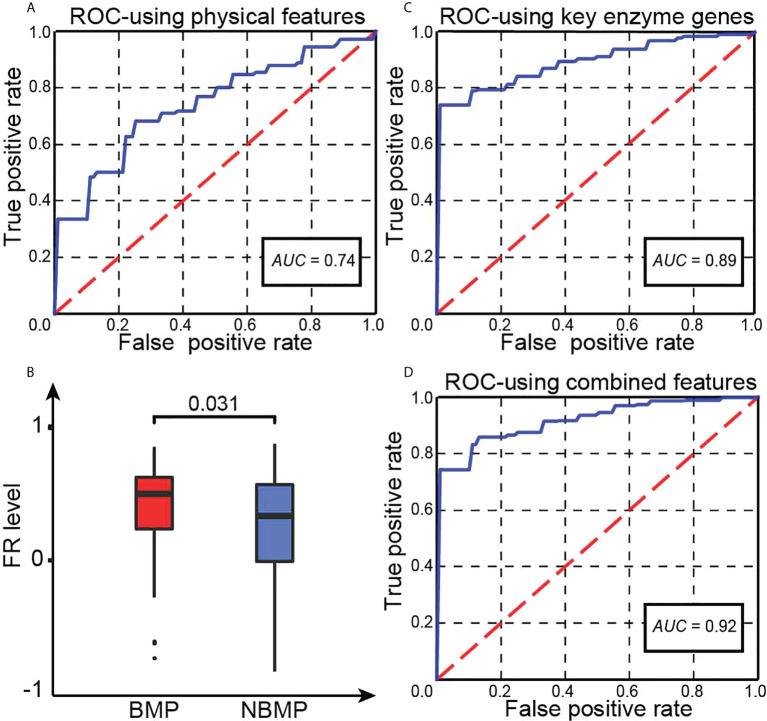
Key statistics. **(A)** The ROC curve of our trained classifier using four physical features. **(B)** Predicted FR levels in BMPs and NBMPs. **(C)** The ROC curve of our second classifier based on the expressions of the 20 selected enzymes. **(D)** The ROC curve of the integrated classifier using the combined features.

We have then conducted a classification analysis between BMPs and NBMPs based on these four features calculated for each sample, using an SVM-based classifier (see Method). A five-fold cross validation shows that this model achieves an *AUC* = 0.74, as shown in **Figure 1A**. This provides strong evidence that the above identified physical features play a key role in dictating if a cancer can metastasize to brain or not. Our next question is: are there other independent features that may further define the characteristics of primary cancer cells that can metastasize to brain or not?

### 3.2 Certain enzymatic functions may dictate if the metastasized cells can survive in brain

While the above analyses are focused on identification of distinguishing cell-surface characteristics of BMPs vs. NBMPs, we study here if the metastasized cancer cells to brain may possess specific functionalities, defined by their activated enzymes, which make the brain-metastasized cells fit well with the brain microenvironment, as Stephen Paget proposed over 130 years ago about cancer metastasis (3).

We have previously demonstrated that most, possibly all, cancers are under persistent intracellular alkalosis, ultimately due to chronical inflammation coupled with iron overload at the tumor sites, which gives rise to a (repeated) inorganic reaction, called *Fenton reaction* (FR): Fe^2+^ + H_2_O_2_ → Fe^3+^ + OH^–^ + HO· to continuously producing OH^-^ when reducing molecules of Fe^3+^ are available around the reaction, which are largely superoxide ( ${\text{O}}_{2}^{-}$ ) released by innate immune cells (21, 30). As response, the affected cells activate a range of reprogrammed metabolisms (RMs) to produce protons collectively at a comparable rate of OH^-^ production by repeated Fenton reactions, which can be rewritten as ${\text{O}}_{2}^{-}+{\text{H}}_{2}{\text{O}}_{2}\to {\text{OH}}^{-}+\text{HO}\cdot {\text{+O}}_{2}$ with Fe^2+^ as a catalyst. Our analyses have revealed that while different cancer cells in TCGA may employ a distinct set of proton-producing RMs, they share a number of RMs, such as *de novo* nucleotide biosynthesis and biosynthesis and deployment of sialic acids (30). The study has provided strong evidence that the distinct set of RMs employed by different subtypes of cancers results in the unique behaviors of individual subtypes of cancers (21, 30, 31).

We have examined the levels of cytosolic FR in BMP and NBMP samples, using the gene set obtained in (21) (**Table S3A**), with results summarized in **Figure 1B**. We note: BMPs have a considerably higher average level of FRs than that in NBMPs (*P*-value< 0.05), revealing that BMPs are facing stronger alkalosis, hence stronger neutralizing RMs.

To identify the set of unique enzymes induced to respond to the alkalosis in BMPs, we have utilized SVM-RFE (see Methods) to conduct a classification along with feature selection between BMPs and NBMPs to identify a minimal set of enzymes whose gene expressions can collectively best distinguish the two sets of tumor samples. Using this method, we have obtained the following 20 most discerning enzymes: *ARSA, CANT1, CASK, CERK, EPHA4, EPHX2, EYA4, EZH2, FMO6P, FYN, GCAT, GLYATL2, MYO3A, NMNAT2, PDE11A, PDE3A, PGLS, ULK1, XDH, YARS1*, out of all the 2,779 expressed enzyme genes (**Table S3B**). The median TPM values of these 20 genes for BMP, NBMP, NBMP-bone, NBMP-liver, and NBMP-lung samples are recorded in **Table S3C**, showing that the BMP group tend to have the highest expressions for most of these enzymes. The subsequent five-fold cross validation of the classification result using the expressions of the 20 enzymes achieves an *AUC* = 0.89, as shown in **Figure 1C**. The functions of the 20 enzymes are summarized in **Table S3D**.

**Figure 2:**
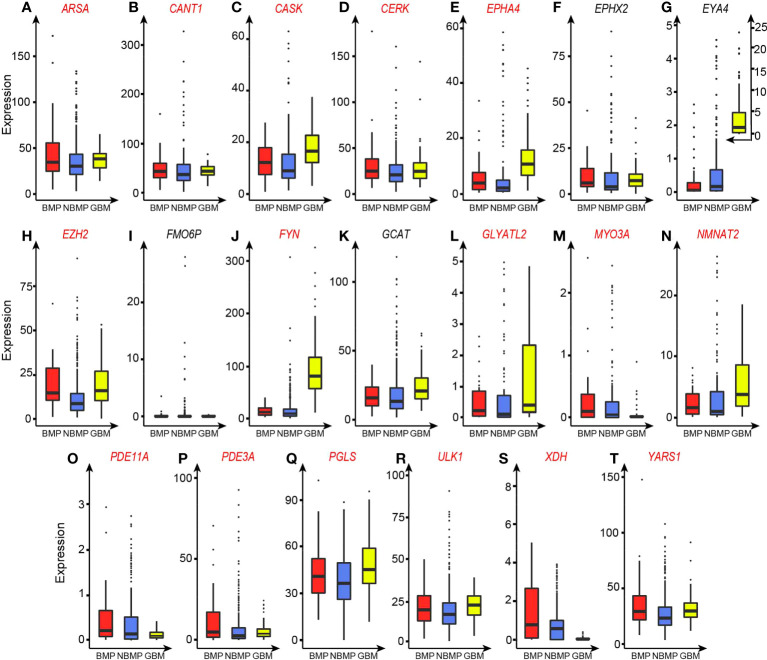
TPM-based expression levels of the 20 selected enzyme genes. Enzymes that catalyze H^+^-producing reactions are marked in red. **(A)** *ARSA*. **(B)** *CANT1*. **(C)** *CASK*. **(D)** *CERK*. **(E)** *EPHA4*. **(F)** *EPHX2*. **(G)** *EYA4*. **(H)** *EZH2*. **(I)** *FMO6P*. **(J)** *FYN*. **(K)** *GCAT*. **(L)** *GLYATL2*. **(M)** *MYO3A*. **(N)** *NMNAT2*. **(O)** *PDE11A*. **(P)** *PDE3A*. **(Q)** *PGLS*. **(R)** *ULK1*. **(S)** *XDH*. **(T)** *YARS1*.

To gain a cellular level understanding about the distinct functions offered collectively by these enzymes, detailed functional analyses are conducted. First, 16 of the 20 enzymes each catalyze H^+^-producing reactions based on the information from Uniprot (15), shown in **Tables 2** and **S3D**, and all the 16 enzymes have higher expressions in BMPs vs. NBMPs (**Figure 2** and **Table 2**). Furthermore, all the 20 enzymes are expressed in brain or the CNS (**Table S3D**) and play roles that could potentially help their host cells to better survive in the brain environment. It is noteworthy that these results highly consistent with the results summarized in **Table S3C** and **Figure 2** as the CNS-originated GBMs have the highest expressions for most of the 20 enzymes.

**Table 2:** H^+^ -producing reactions catalyzed by 16 selected enzymes.

Enzymes	MedianTPM-BMPs	MedianTPM-NBMPs	H^+^ -producing reactions
*ARSA*	34.61	30.21	H2O + N-acyl-1-β-D-(3-O-sulfo)-galactosyl-sphing-4-enine → a β-D-galactosyl-(1↔1’)-N-acylsphing-4-enine + **H^+^** + sulfate
*CANT1*	41.95	37.17	a ribonucleoside 5’-diphosphate + H2O → a ribonucleoside 5’-phosphate + **H^+^** + phosphate
*CASK*	12.34	8.89	ATP + L-seryl-[protein] → ADP + **H^+^** + O-phospho-L-seryl-[protein]
*CERK*	25.48	19.19	an N-acylsphing-4-enine + ATP → ADP + an N-acylsphing-4-enine 1-phosphate + **H^+^**
*EPHA4*	3.89	1.74	ATP + L-tyrosyl-[protein] → ADP + **H^+^** + O-phospho-L-tyrosyl-[protein]
*EZH2*	14.61	8.53	L-lysyl27-[histone H3] + 3 S-adenosyl-L-methionine → 3 **H^+^** + N6,N6,N6-trimethyl-L-lysyl27-[histone H3] + 3 S-adenosyl-L-homocysteine
*FYN*	11.32	9.37	ATP + L-tyrosyl-[protein] → ADP + **H^+^** + O-phospho-L-tyrosyl-[protein]
*GLYATL2*	0.15	0.06	an acyl-CoA + glycine → an N-acylglycine + CoA + **H^+^**
*MYO3A*	0.10	0.04	ATP + L-seryl-[protein] → ADP + **H^+^** + O-phospho-L-seryl-[protein]
*NMNAT2*	1.62	0.97	diphosphate + NAD+ → **H^+^** + ATP + β-nicotinamide D-ribonucleotide
*PDE11A*	0.17	0.13	3’,5’-cyclic GMP + H2O → GMP + **H^+^**
*PDE3A*	4.35	1.94	a nucleoside 3’,5’-cyclic phosphate + H2O → a nucleoside 5’-phosphate + **H^+^**
*PGLS*	40.61	36.71	6-phospho-D-glucono-1,5-lactone + H2O → 6-phospho-D-gluconate + **H^+^**
*ULK1*	19.70	17.45	ATP + L-seryl-[protein] → ADP + **H^+^** + O-phospho-L-seryl-[protein]
*XDH*	0.79	0.58	H2O + NAD+ + xanthine → **H^+^** + NADH + urate
*YARS1*	29.19	23.15	ATP + L-tyrosine + tRNATyr → AMP + diphosphate + **H^+^** + L-tyrosyl-tRNATyr

Colored text is laying the emphasis to the ‘production of H^+^’ in the Enzymatic reactions.

Published studies suggest that brain-metastasizing tumor cells need to alter their metabolisms to gain brain-like and neuron-like properties for brain metastasis (13, 32–34). For example, brain metastasized tumor cells need to overcome stressors from the antimetastatic behaviors by reactive astrocytes in brain CNS, through increasing their brain-like and neuron-like properties to evade attacks from the reactive astrocytes and even to transform to chemo-protectors (7, 13, 33). In addition, metabolic changes have been demonstrated to serve as the foundation for enhanced cancerous cell division and survival in brain CNS (32).

We have checked the functions of the 20 enzymes **(** **Table S3D**). *ARSA*, a regulator of neuron myelination, can boost the neuronal survival and differentiation (35). *CASK* regulates the development of brain neurons (36), hence providing a range of capabilities for cell survival. *CERK* is highly expressed in cerebellar Purkinje cells and converts ceramide to a sphingolipid (37), which is known to play powerful roles in dealing with oxidative stress (38). *EPHA4* meditates motor neuron death and regulates axon guidance and proliferation of neural stem cells (39), hence having the capabilities for overcoming stresses. *EPHX2* alters neuronal susceptibility to ischemic cell death (40), another survival related gene. *EZH2* is important for neuronal survival and regulates the self-renewal and differentiation of cells in the cerebral cortex (41, 42). *FYN* tempers excitatory and inhibitory synaptic transmission stimuli of neurons (43) and is a key responder to oxidative stress (44). *NMNAT2* acts as an essential axon maintenance factor of neurons (45) and its overexpression can ameliorate oxidative stress (46). *PDE11A* is highly expressed in hippocampus and plays key roles in inflammation modulation (47). *ULK1* works in brain iron accumulation and regulates the autophagy-mediated cell survival of brain-metastasized tumor cells (48). *XDH* is needed for neuronal survival *via* maintenance of cold tolerance (49). *PGLS* is a marker gene for brain-derived cells, associated with cell redox homeostasis, and regulates the viability of breast tumor cells in the brain microenvironment (32). These indicate that all these genes have capabilities in cell survival, stress adaption, and inflammation management. In addition, literature reviews show that most of these enzymes have been reported to be relevant to cancer metastasis, especially brain metastasis, as summarized in **Table S3D**.

To further investigate the functions of these 20 enzymes in BMPs, we have conducted a co-expression analysis between the 20 enzymes genes and all the other expressed enzyme genes in BMPs, and obtained 27 additional enzyme genes that strongly co-express with at least one of the 20 genes (**Table S3E**), which is followed by a pathway-enrichment analysis over the 47 genes, giving rise to 170 upregulated pathways (no downregulated pathway are obtained), listed in **Table S3F**.

We note from the table that the 170 pathways fall largely into two groups, cell-cycle related pathways (74/170) and metabolic pathways (58/170). The enriched cell-cycle activities are consistent with the published studies reporting that brain metastases generally require highly increased cell-cycle activities (32–34). Out of the 58 metabolic pathways, 27 are related to nucleotide metabolism; 8 are relevant to epigenomic activities, such as histone modification and methyltransferase; 13 related to phosphorylation/dephosphorylation; and 10 are involved in second messenger signaling.

Our previous study has established that nucleotide biosynthesis is the most powerful proton-producer (21), which is induced by vast majority, possibly all cancers in TCGA, to neutralize the persistently produced OH^-^ by FRs; and the rate of cell division is predominantly dictated by the rate of nucleotide biosynthesis (30), hence the observed increased nucleotide metabolism is consistent with the elevated cell-cycle progression and faster cell division.

The higher levels of epigenomic activities suggest that more stress-response activities are induced in the BMPs compared to NBMPs. It is well known that the increased epigenomic activities tend to be induced to assist the host cells to cope with persistent and severe stressors (50). Therefore, cells in BMPs are prepared better to cope with new stressors in the brain microenvironment.

Furthermore, it has long been observed that cancers tend to overexpress a large number of kinases (51), which is a result of alkalosis-responding metabolic reprogramming as each phosphorylation act creates one net proton (30). The increased phosphorylation will activate more enzymes, which makes the overall metabolism more active. We postulate that the more activated enzymatic functions in BMPs will equip the host cells with more capabilities to overcome the new stressors in the brain environment.

The cAMP and cGMP signaling system is a key second messenger and involved in a wide range of cellular functions. This system is mostly repressed in primary cancers since it involves the E3 ubiquitin ligases, which are known to be generally repressed in cancer (52, 53). The unique feature of upregulated cAMP and cGMP signaling may make the host cells more adaptable to the stressful CNS microenvironment (54, 55). The unique feature of BMPs over-expressing and utilizing the system may give the host cells a powerful way to utilize a wide range of cellular functions, which may be limited in other primary cancer cells.

Overall, our analyses have revealed that the BMPs have a unique collection of functional capabilities that can help the host cells to better survive the brain microenvironment.

### 3.3 Prediction of BMPs based on transmembrane proteins and intracellular enzymes

We have conducted a classification prediction between the BMP and NBMP samples based on the physical features as shown in Equations (6) - (9) along with the expression data of the 20 selected enzymes, using an SVM model. Five-fold cross validation shows that the model achieves *AUC* = 0.92, as shown in **Figure 1D**. This *AUC* is higher than individual models trained on the physical features and on the enzyme expressions, separately, hence indicating that information from both categories contributes to the classification performance between BMPs and NBMPs.

Based on this, we predict that the physical features define largely the types of metastasizing cells can go through the BBBs to reach the internal parts of a brain and the enzymes may define the characteristics of primary cancer cells that survive and thrive in the brain microenvironment. Compared with published studies on prediction of brain metastasis for primary tumors, our model shows higher or comparable accuracy (56, 57), but has two key superiorities: (i) the features used in our model are strongly supported by published studies and provide strong information about the key mechanisms of brain metastasis; and (ii) the primary locations of BMPs are not limited to one organ instead any organs, strongly suggesting that our predicted mechanism captures something fundamental.

## 4 Discussion

Through modeling of the extracellular amino acid sequences of transmembrane proteins and gene-expressions of specifically expressed intracellular enzymes in BMPs, we have identified discerning features that can well distinguish BMPs from NBMPs, from which we have predicted the distinguishing characteristics of BMPs, namely (i) the physical features of the expressed transmembrane proteins that BMPs should have; and (ii) the expression patterns that specific enzymes should have. Our predictions are highly consistent with a wide range of published literature and extend the current model of BMP cancers. In addition, a key novelty of this work is that we have examined the question of brain metastasis at the basic physics and chemistry level, rather than the typical approaches that study cancer biology at the biomolecular levels, namely signaling, mutations and regulatory events. We anticipate that the approach used here should be applicable to elucidation of molecular basis for other cancer subtyping problems, including metastases to other organs, cancers of different levels of malignancy, cancers with distinct levels of drug resistance among possibly others.

Information gained here could possibly applied to drug target identification for stopping brain metastases through inhibiting the most contributing transmembrane proteins and/or intracellular enzymes in BMPs *via* drug repurposing as multiple proteins and enzymes identified in our study have known drugs, such as Amitriptyline for protein *OPRD1*, Suramin for enzyme *ARSA*, and Tazemetostat for enzyme *EZH2* (15).

Further improvement will involve quantification of the statistical and qualitative models developed here, which has limited the level of resolution of our results. For example, our analyses have revealed that a key property of the identified enzymes in BMPs is that they mostly catalyze H^+^-producing reactions. However, we could not inform the relative levels of H^+^ productions by different enzymes in BMP cancer cells, while such information could prove to be essential for drug target identification for possibly stopping brain metastases. One possible way to make our models more quantitative is through representing the models as a set of Michaelis-Menten equations as done in our previous studies (21, 58).

## Data availability statement

The original contributions presented in the study are included in the article/**Supplementary Material**. Further inquiries can be directed to the corresponding author.

## Author contributions

DL is for design of methods, programming, paper writing and revising. JB is for design of methods and programming. YX is for design of methods and paper writing and revising. QC, RT, ZA, JX, and YQ are for data collection and processing. All authors contributed to the article and approved the submitted version.

## Acknowledgments

The correspondence author thanks Georgia Research Alliance for continuous support to his lab since 2003.

## Conflict of interest

The authors declare that the research was conducted in the absence of any commercial or financial relationships that could be construed as a potential conflict of interest.

## Publisher’s note

All claims expressed in this article are solely those of the authors and do not necessarily represent those of their affiliated organizations, or those of the publisher, the editors and the reviewers. Any product that may be evaluated in this article, or claim that may be made by its manufacturer, is not guaranteed or endorsed by the publisher.
